# End-of-Life Care for Neonates: Assessing and Addressing Pain and Distressing Symptoms

**DOI:** 10.3389/fped.2020.574180

**Published:** 2020-09-24

**Authors:** Shelly Haug, Alicia Dye, Sara Durrani

**Affiliations:** ^1^Department of Neonatology, Eastern Idaho Regional Medical Center, Pediatrix Medical Group, Idaho Falls, ID, United States; ^2^Department of Pharmacy, Eastern Idaho Regional Medical Center, Idaho Falls, ID, United States; ^3^Department of Neonatology, Beth Israel Deaconess Medical Center, Boston, MA, United States

**Keywords:** neonatal, end-of-life care, neonatal hospice, neonatal palliative care, neonatal pain

## Abstract

One of the most essential components of end-of-life (EOL) care for neonates is assessing and addressing distressing symptoms. There is limited evidence to guide neonatal EOL symptom management and therefore significant variety in treatment (1–4). EOL neonatal palliative care should include identifying and relieving distressing symptoms. Symptoms to manage at neonatal EOL may include pain using both non-pharmacologic and pharmacologic comfort measures, respiratory distress, secretions, agitation and neurologic symptoms, nutrition and gastrointestinal distress, and skin care. Also of equal importance is communication surrounding familial existential distress and psychosocial care (1, 5–7). Institutions should implement a guideline for neonatal EOL care as guidelines have been shown to decrease variability of interventions and increase use of pharmacologic symptom management (4). Providers should consult with palliative care teams if available for added multidisciplinary support for family and staff, which has been shown to enhance EOL care in neonates (8, 9).

## Introduction

Neonatal end-of-life (EOL) care includes addressing pain and other distressing symptoms (1, 7, 10, 11). Neonatal intensive care units (NICUs) are generally competent in EOL care of neonates; however, evidence shows there are wide variations in methods for evaluating and addressing symptoms (1, 12). This variation likely stems from limited evidence regarding neonatal EOL symptom management given the lack of on-label neonatal pharmacologic treatments (5, 10). A 2016 survey showed only 55% of NICUs in the United States have a neonatal EOL guideline, and 45% do not have access to a palliative care team, showing there is much room for improvement regarding neonatal EOL care (1). This review aims to incorporate scientific research evidence with clinical expertise to aid in assessing and addressing neonatal EOL symptoms.

### Vignette

*A 25-week gestation 5-day-old neonate with severe grade 4 bilateral intraventricular hemorrhage and acute pulmonary hemorrhage is currently intubated on high frequency oscillator ventilation. Parents have decided to transition to EOL comfort care. The bedside nurse is concerned regarding increased facial grimacing of the neonate and asks which pain scale to use*.

## Assessment of Pain in Neonates

The most common symptom related to pediatric EOL care is pain, which is often underestimated in neonates (13–15). In comparison to adults, neonates experience pain that is more severe, diffuse, and prolonged (16). Several different pain assessment tools are validated for neonates; however, one has not been found superior over the others (5, 16, 17). These assessment tools do not evaluate for distress from other etiologies such as hunger, and interpretation must be completed in proper clinical context (5).

Pain should be evaluated every 15 min upon initial discovery. Once controlled, we recommend evaluation of pain at least every 3 h. The scale chosen should be part of your institutional guidelines. Your chosen assessment scale should be used consistently, as scenarios allow, and chosen with input from your nursing staff. Assessment scales to consider include but are not limited to the following.

Acute neonatal pain scales to consider:

- Behavioral Indicators of Infant Pain (BIIP) evaluates pain using behavioral state, facial expressions, and hand movements. Validated since 2007 for acute pain in preterm infants 23- to 32-weeks gestation (18).
- Premature Infant Pain Profile (PIPP) uses gestational age, behavior, heart rate, oxygen saturation, brow bulge, eye squeeze, and nasolabial furrow. Validated since 1996 for acute and postoperative neonatal pain (19).

Prolonged neonatal pain scales to consider:

- COMFORTneo Scale uses alertness, calmness, respiratory response, crying, body movement, facial tension, and body muscle tone. It was validated in 2009 for prolonged neonatal pain (20).
- EDIN Scale (Echelle Douler Inconfort Nouveau Né) evaluates facial activity, body movements, quality of sleep, quality of contact with nurses, and consolability. Validated in 2001 for prolonged pain specifically in preterm neonates (21).
- NPASS (Neonatal Pain, Agitation, and Sedation Scale) looks at crying, behavior, facial expression, tone, and vital signs. Validated in 2008 for prolonged postoperative pain as well as pain with mechanical ventilation. It is validated for use down to 23-weeks gestation (22).

### Vignette Discussion

*Does your unit have a consistent acute pain scale used at EOL? We recommend BIIP, PIPP, or NPASS scale here given the relatively acute nature, prematurity, and use of mechanical ventilation. No one scale has been found superior, though certain scales are validated for particular scenarios such as prolonged pain or gestation*.

## Non-Pharmacological Comfort Measures

Critically ill neonates frequently undergo painful interventions (16, 23). Care should be given to decrease noxious stimuli. Regularly scheduled procedures, such as bath time, can often be held or deferred; orders for obtaining vitals less often can decrease noxious stimuli (24).

Non-pharmacological neonatal comfort measures include swaddling, facilitated tucking, kangaroo care/skin-to-skin care, non-nutritive sucking with or without oral sucrose, and breastfeeding (16, 25, 26). Each of these methods has been shown to increase comfort or decrease oxidative stress markers of neonates in situations of acute pain. In fact, combining measures enhances comfort over using a single method alone (5, 27).

## Pharmacological Management

**Figure 1:**
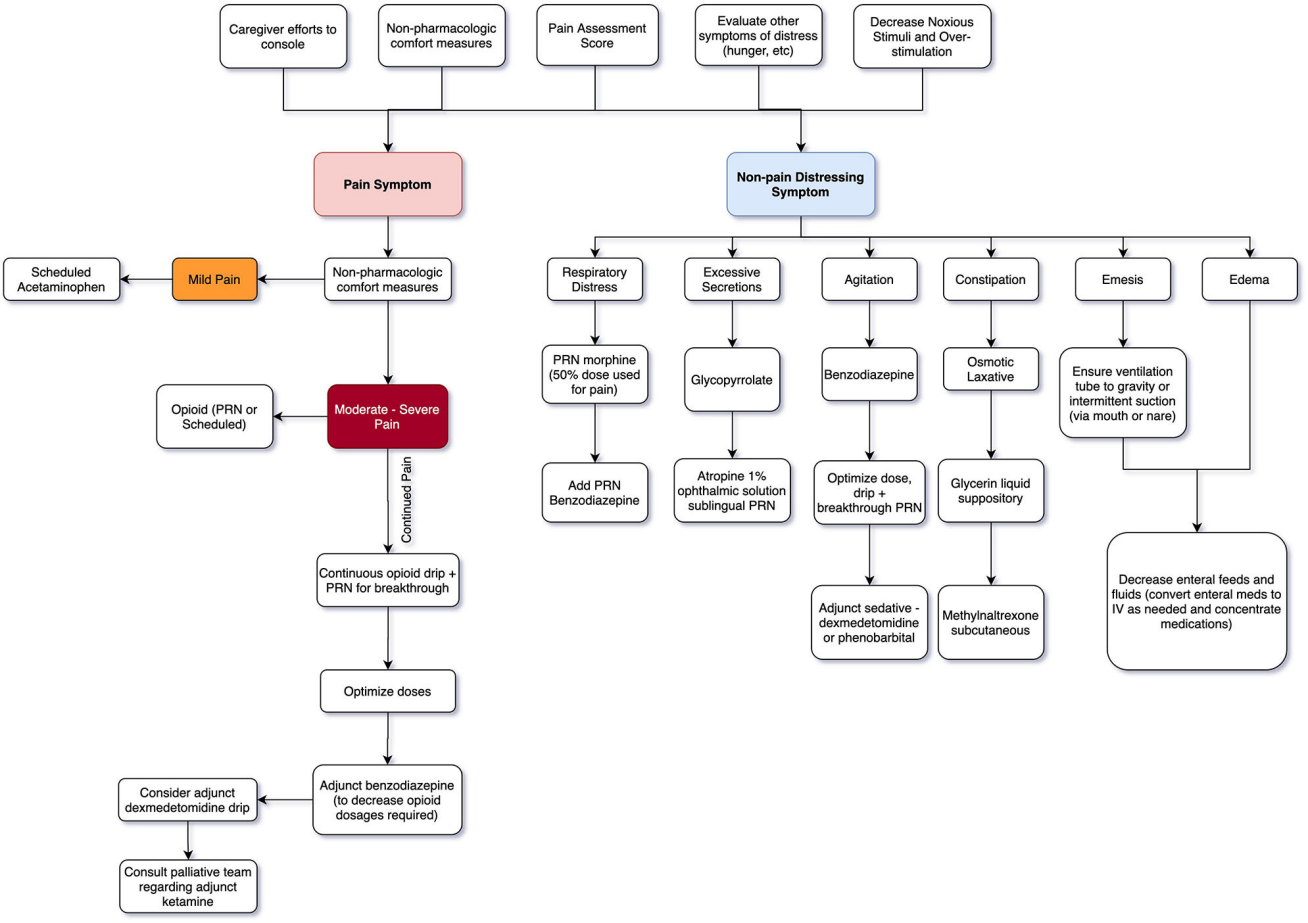
Neonatal EOL Symptom Algorithm.

Non-opioid mediations are recommended for mild pain, while opioid medications with or without adjuvant therapies are used for moderate to severe pain (16, 24). Efficacious administration routes include enteral, intravenous (IV), mucosal (buccal, lingual, or rectal), intranasal, transdermal, and subcutaneous depending on the agent. Agents may need to be combined for optimal comfort (5, 16). See Table 1 for initial dosing guidelines and Figure 1 for a general guide to stepwise approach for providers.

**Table 1:** Neonatal pharmacologic agents for end-of-life palliative care with initial doses*.

	**Enteral or Mucosal**	**Intravenous**	**Other**
**NON-OPIOIDS**			
Sucrose	Buccal/Lingual: 0.1–1 mL/dose every 2 min		
Acetaminophen	PO: 10–15 mg/kg every 6–8 h	7.5–15 mg/kg every 6–8 h	
	PR: 15–20 mg/kg every 6–8 h		
**OPIOIDS**			
Morphine	PO/PR/Buccal: 0.1–0.2 mg/kg every 3–6 h	Bolus: 0.05–0.1mg/kg every 2–4 h	SC: 0.1–0.2 mg/kg every 3–6 h
		Continuous: 0.01–0.1 mg/kg/h	
Fentanyl	Buccal: 1–2 mcg/kg every 1–4 h	Bolus: 1–3 mcg/kg every 5–15 min	Intranasal: 1–2 mcg/kg every 1–4 h
		Continuous: 0.5–5 mcg/kg/h	
**ADJUVANTS**			
Midazolam	PO/Lingual/PR: 0.2 mg/kg every 2–4 h	Bolus: 0.05–0.1 mg/kg every 2–4 h	Intranasal: 0.1–0.3 mg/kg every 2–4 h
		Continuous: 0.03–0.06 mg/kg/h	
		Maximum Continuous: 0.3–0.5 mg/kg/h**	
Lorazepam	PO/PR/Buccal: 0.05–0.1 mg/kg every 6–8 h	Continuous: 0.05–0.1 mg kg every 6–8 h	
Dexmedetomidine		Bolus: 0.5–1 mcg/kg/h	
		Continuous: 0.3–1 mcg/kg/h	
Clonidine	PO: 2–4 mcg/kg every 4–6 h	***	
Phenobarbital	PO/PR: 5 mg/kg every 24 h	Bolus: 5 mg/kg every 24 h	

*IV, intravenous; PO, by mouth; PR, rectal; SC, subcutaneous*.

* *Table lists suggested doses, recommend titrating as required for symptom management*.

** *Maximum sedation EOL dosing, also with anticonvulsant activity at this level which may allow other anticonvulsants to be discontinued*.

*** *No IV formulation available, see dexmedetomidine IV*.

The principle of “Double Effect” is an important consideration for clinicians concerned about the use of pharmacologic agents for EOL care. The principle asserts it is morally permissible to unintentionally cause harm as a side effect if the original overarching intent is to bring about a good effect. Extrapolating this principle to EOL care explains the use of medications that might potentially cause respiratory depression given the intent is to relieve pain and suffering (28, 29).

### Non-opioid Medications

Sucrose is effective in reduction of pain behavior during acute procedures and noted to have increased efficacy when used in combination with nonnutritive sucking (5, 16, 30).

Although acetaminophen does not show analgesic effectiveness for reducing effects of painful procedures in neonates, it does show an opioid sparing effect (31, 32). Acetaminophen should only be used to treat mild pain or as an opioid sparing agent. Side effects include liver toxicity, although this is less common in neonates than older children (33).

Literature examining nonsteroidal anti-inflammatory agents such as ibuprofen, ketorolac, or indomethacin for neonatal EOL care is lacking and therefore not generally recommended.

### Opioids

Opioids should be first-line therapy in management of neonates with moderate to severe pain. Route of administration should take into consideration onset of action, duration of action, and most efficacious route for desired opioid.

Morphine has been shown to be less effectively absorbed when used mucosally due to less lipophilicity than other opioids like fentanyl (34, 35). Side effects include urinary retention, decreased gastrointestinal motility, nausea, vomiting, hypotension, and respiratory depression. Respiratory depression is always preceded by sedation (16, 36).

Benefits of fentanyl include fast onset of action and efficacy of intranasal administration due to high lipophilicity (16, 34). Fentanyl also does not cause histamine release; therefore, it has decreased vasomotor center activity causing less hypotension than morphine. It also has a shorter duration of action than morphine. A notable adverse effect is chest wall rigidity, especially with higher doses and rapid IV administration (16).

### Benzodiazepines

Benzodiazepines potentiate inhibitory GABA (gamma-aminobutryric acid) neuronal activity of the central nervous system (37). They are potent sedative and anxiolytic medications without analgesic effect, which can decrease need for opioids (16). Midazolam has rapid onset as well as short duration of action, enabling ease of titration (5). Lorazepam has an intermediate onset of action but with longer duration of action facilitating baseline sedation needs (37). Side effects include myoclonic jerking and hypotension (16).

### Alpha 2-adrenoreceptor Agonists

Dexmedetomidine and clonidine are alpha2-adrenoreceptor agonists that have sympatholytic, sedative, and analgesic effects. In contrast to opioids, they do not have significant ventilatory effects. The most notable side effects include bradycardia and hypotension (5, 16).

### Alternative Sedation Medications

Phenobarbital is a barbiturate that can be an adjunct medication for palliative sedation (38–40). It has sedative and anxiolytic effects without analgesia via its inhibition of the central nervous system through augmenting the GABA system. The preferred route in this context is oral due to slow absorption causing less severe side effects (5). Dosage when used as an adjunct sedative is much less than for seizure control in neonates (see Table 1). Clinicians should use caution if combining with opioids or benzodiazepines as this may increase the risk of respiratory depression. Pentobarbital is another barbiturate with shorter half-life than phenobarbital but is not well studied in end-of-life care for neonates (16).

### NMDA-Receptor Antagonist

Ketamine is an N-methyl-D-aspartate (NMDA)-receptor antagonist used to decrease central sensitization to painful stimuli and perception of pain. Ketamine has proven effective in neonates for peri-procedural sedation and analgesia with a short duration of action. It should only be used in combination with a sedative medication due to hallucination risk. Adjunct dose to consider is 0.5–2 mg/kg/dose intravenous although use at end of life is not well-studied (16, 41).

### Vignette Discussion

*What comfort medications you consider depend on distressing symptomatology for infant and family. Is the neonate already on an opioid drip for painful chest tubes, ventilator, or recent surgical procedure? At times you may only need titration for improved effect. In other cases, your main distressing symptom may be respiratory distress or secretion management without any pain issues. See* *Figure 1*.

## Respiratory Symptom Management

### Respiratory Distress

EOL respiratory distress may manifest as retractions, tachypnea, grunting, nasal flaring, or gasping. Non-pharmacologic treatment interventions may include positioning modifications such as elevating the head of the bed and positioning side-lying or prone. Use of a fan with air movement toward the patient's face has been shown to relieve dyspnea symptoms in adult hospice patients (42). Use of oxygen is not beneficial for respiratory distress for most patients at EOL (43, 44).

Pharmacologic management options include opioids, which can reduce central sensitivity to hypoxia and hypercapnia (45). Morphine is commonly used in neonates and is usually effective at 50% of the dose used for pain (see Table 1). Use of an opioid with a benzodiazepine has been shown to significantly reduce respiratory EOL symptomatology (16, 45).

### Excessive Secretions

Inability to swallow saliva may lead to pooling in the posterior pharynx and noisy breathing. Anticipatory guidance with the family should be based around treating only if symptoms becomes distressing to the neonate. Least invasive non-pharmacologic treatment would first include decreasing or discontinuing hydrating fluids in an effort to decrease secretion production, positioning the infant to allow gravity to drain secretions (i.e., side-lying or prone), and gentle shallow oral suctioning with soft catheter as needed (46).

Atropine 1% ophthalmic solution may be given sublingually 1–2 drops every 1–6 h as needed (5, 16, 45, 47). Atropine inhibits local salivation via anticholinergic activity. Atropine sublingual drops have shown reasonable effect in reduction of terminal respiratory secretions while not showing cardiac or central nervous system symptoms, though most evidence is adult based at this time (48).

Glycopyrrolate is an anticholinergic that reduces gastric, pharyngeal, tracheal, and bronchial secretions. Dosing PO is 20–100 mcg/kg/dose every 4–8 h as needed. Dosing IV or subcutaneous is 2–10 mcg/kg/dose every 4–8 h as needed. If pharmacologic intervention is needed, glycopyrrolate is generally the preferred pediatric anticholinergic option as it does not cross the blood-brain barrier, so it rarely has central side effects (49). Side effects include mucous plugging due to thickened secretions.

Scopolamine transdermal patches show no increased effectiveness in secretion management and are usually avoided in neonates due to risk of central side effects from crossing the blood brain barrier (50).

### Compassionate Extubation

Clinicians must be cautious in the setting of compassionate extubation or discontinuation of respiratory support in neonates. Experts in neonatal EOL care recommend initiating opioids with or without synergistic benzodiazepine and then a slow wean of ventilator settings while monitoring for development of respiratory distress. This allows for titration of medication for symptoms prior to extubation. Parents should be involved with the non-pharmacologic comfort role if able, including holding, swaddling, rocking, and positioning for secretions. Anticipatory guidance should be given to parents in monitoring for pain and respiratory symptoms. Anticipation and prevention of pain, agitation, and dyspnea symptoms is paramount to decrease familial and neonatal discomfort (5, 16).

## Agitation and Neurologic Symptom Management

There are multiple etiologies for agitation during EOL care for neonates, which may include brain injury, malformations, seizures, or iatrogenic neonatal abstinence syndrome due to long-term drug exposure from hospitalization. Non-pharmacologic interventions should not be employed first in neurologic scenarios such as seizures.

Pharmacologic interventions for agitation include benzodiazepines as well as opioids as they have a synergistic sedation effect. Barbiturates may also be considered (29). See Table 1 for agitation medication recommendations. It is important to balance over-sedation and lethargy with desired control of symptoms. Seizure control should include phenobarbital 20 mg/kg/dose up to 40 mg/kg/load IV. Medical consultation with your pediatric neurologist can guide further anticonvulsant recommendations.

## Nutrition and Gastrointestinal Distress Symptom Management

### Nutrition

An important component of neonatal end-of-life care is addressing artificial hydration and nutrition as well as natural hydration/nutrition. There is no reason to preclude breastfeeding or bottle feeding as part of comfort during EOL care if the neonate orally feeds and doing so does not exacerbate distress.

However, discussions around withdrawal of artificial hydration and nutrition present a challenge in neonatal EOL care. Culturally, many parents attach meaning and symbolism of caring for their neonate with nutrition. Using clear language during discussions is of utmost importance (16). Receiving artificial hydration and nutrition may not be in the best interest at EOL as it may worsen symptomatology through fluid overload, respiratory distress, increased abdominal distention, and discomfort or nausea and vomiting (5). Addressing subsequent side effects of dehydration is also important, for example, oral hygiene (see Mouth Care section below) (16).

### Constipation

Opioid induced constipation is multifactorial including decreased gastrointestinal motility, inhibition of mucosal transport of electrolytes and fluids, as well as interference with the defecation reflex (16). The risk of opioid-induced constipation increases with duration of opioid therapy. Laxative treatment such as polyethylene glycol is considered first-line with neonatal doses of 0.2–0.8 g/kg/day (51–53). Liquid glycerin suppository 0.2 mL per rectum may be given in addition to scheduled osmotic laxative.

A last resort treatment consideration is methylnaltrexone for reversal of some opioid side effects without precipitating withdrawal. Methylnaltrexone is a peripheral acting mu-opioid receptor antagonist that has emerging data in neonates. Neonatal dosing used is 0.15 mg/kg subcutaneous once daily until bowel movement occurs (54, 55).

## Skin, Eye, and Mouth Care

Physiological changes during EOL may compromise the skin and soft tissues manifesting as changes in skin (color, elasticity, or integrity) or as subjective symptoms such as pain or itching (56). Areas of decreased cutaneous perfusion may exhibit as dusky erythema, mottled discoloration, or areas of localized cooling. Care should include skin assessments and prevention of excessive pressure, friction, moisture, and immobilization during EOL care. Clinicians should monitor for incomplete eyelid closure as this increases risk of exposure keratopathy, which might cause ophthalmic pain and sensitivity (57). Care of mouth xerostomia should also be part of EOL assessment care.

Evidence based treatments for neonatal EOL skin care are lacking; however, pediatric studies show topical emollients may aid in reduction of cutaneous dryness (58). If incomplete eyelid closure is noted, artificial tear ointments have shown to be helpful in multiple intensive care settings (59). Moist sterile water swabs work well for neonatal xerostomia care (60, 61). Topical petroleum jelly to dry lips may be helpful as well (60). Parental involvement in symptom assessment has been shown to be a predictor of quality EOL symptom treatment (62). If counseled appropriately, these skin, eye, and mouth changes can easily be assessed by parents and treated as needed (63).

## Familial Existential Distress and Psychosocial Care

Providers should be mindful of the propensity for family members to feel enormous guilt and question their decisions. Many families will be in existential crisis and openly question why this is happening. This part of the grief process should be normalized. Some parents may need reassurance that they are in fact a good advocate for their baby while also being present for their neonate's EOL (11).

Some families have moral distress in believing that withdrawing technological support is not permissible in their cultural or religious customs. Reframing discussions in a way that the family does not have to decide anything may be helpful. For example, the physician may say something like “Additional technological support is inappropriate and will increase the infant's suffering. I recommend we increase the palliative care services and work intensively now to support comfort for your infant” (11).

Helpful discussions for family may include openly asking about religious rituals such as baptism that may be important for closure. Consultations to pastoral care or clergy that align with their religious preference may be of comfort during EOL transition. Consider involvement of social work, child life, and palliative medicine if available at your hospital and circumstances allow. Developing a unit checklist for bedside nurses may aid families in obtaining memories with mementos such as a lock of hair, hospital name bands, crib cards, and photographs (10, 11). Families should also be given bereavement and counselor contact information for the area to aid the grief and healing process after perinatal loss (10, 64, 65).

Anticipatory guidance is an important part of the bereavement process for women who are lactating. Consider consulting your lactation specialist. Helpful methods of care may include the use of medications such as estrogen containing birth control pills, a process of decreasing frequency of milk expression without completely emptying the breast, or milk donation for a time (66, 67).

## Institutional Neonatal EOL Care Guidelines

Most institutions across the United States do not formally address neonatal EOL care in a guideline format (1). However, implementation of clinically practical guidelines increases consistency in patient care as well as education and comfort of staff/trainees (4, 38, 68, 69). Neonatal EOL guidelines should be individualized by institution and case to case as necessary.

## Discussion

One of the most difficult parts of neonatal EOL care includes discussion with family. Acknowledgment should be a tool we use with our families, staff, and ourselves. Acknowledge that EOL is sometimes unfair and difficult, and unimaginable even. Acknowledge it is natural to want to lessen the blow of information you have to discuss as the provider, despite research showing this does not help (70). Acknowledge the difficulty of family needing to digest what you discuss. Share with the family how much you wish the situation were different. Speak information as clearly and succinctly as possible. Answer questions, attempt to anticipate questions, and normalize their concerns (71).

EOL care is a multifaceted approach including identifying and treating EOL symptoms while also addressing familial existential distress and psychosocial care (1, 5–7). Implementation of a guideline for neonatal EOL care at your institution can aid in improved EOL processes (4). Consulting with neonatal palliative care teams, if available, can add important multidisciplinary support that enhances neonatal EOL care (8, 9). Increasing neonatal EOL care palliative education with trainees, nursing staff, and colleagues at your institution can increase comfort and competence with EOL care (72). In all, EOL care is a sacred and deeply meaningful part of life around the world. By assessing and addressing symptoms as well as promoting a culture of respect surrounding familial or spiritual traditions, we can help families find closure with a dignified and peaceful neonatal EOL process (73).

## Author Contributions

SH collected and analyzed data as well as primarily wrote the manuscript. AD and SD critically reviewed and contributed to the final draft of the manuscript. All authors contributed to the article and approved the submitted version.

## Conflict of Interest

The authors declare that the research was conducted in the absence of any commercial or financial relationships that could be construed as a potential conflict of interest.
